# Methicillin-resistant *Staphylococcus aureus* Necrotizing Pneumonia

**DOI:** 10.3201/eid1110.050776

**Published:** 2005-10

**Authors:** Monica Monaco, Rosa Antonucci, Paolo Palange, Mario Venditti, Annalisa Pantosti

**Affiliations:** * Istituto Superiore di Sanità, Rome, Italy; † Università La Sapienza, Rome, Italy

**Keywords:** Staphylococcus aureus, Panton-Valentine leukocidin, pneumonia, infections, letter

**To the Editor:** Methicillin-resistant *Staphylococcus aureus* (MRSA) strains account for >40% of all hospital-acquired *S. aureus* infections in Italy (*1*). Although cases of community-acquired MRSA (CA-MRSA) infections have been reported in recent years (*2*), these isolates have not been characterized for Panton-Valentine leukocidin (PVL) (*3*); therefore, the presence of isolates with the typical characteristics of CA-MRSA (*4*) in Italy remains unknown.

At the beginning of April 2005, a 37-year-old woman was admitted to the University Hospital Policlinico in Rome because of fever, cough, and headache. Her medical history was unremarkable. She was a teacher in a school for foreign students in Rome, smoked 3 cigarettes per day for 15 years, and reported no recent travel abroad. Her 5-year-old daughter had influenzalike symptoms in the previous week. At hospital admission, her temperature was 39°C, heart rate was108 beats/min, respiratory rate was 32 breaths/min, and blood pressure was 105/70 mmHg. Arterial blood gas analysis showed mild hypoxemia and hypocapnia (PaO_2_ 73 mm Hg and PaCO_2_ 34 mm Hg on room air). Leukocyte count was 24,360 cells/μL (81% polymorphonuclear cells), and platelet count was 506,000/μL. Chest radiograph showed infiltrates in the right upper and lower lobes and left lower lobe. Empiric treatment with clarithromycin and ceftriaxone was started, but the patient's clinical conditions did not improve. Culture of sputum samples obtained at admission yielded growth of MRSA. Computed tomographic scan showed multiple lung cavitary lesions, indicating necrotizing pneumonia. On day 3 of admission, antimicrobial drug therapy was changed to linezolid (600 mg 2 times a day). Fever resolved, and the patient's condition rapidly improved. The patient was discharged after 14 days of linezolid treatment. At discharge, leukocyte count was 6,040 cell/μL (58% polymorphonuclear cells), and arterial blood gas analysis showed PaO_2_ of 88 mm Hg.

The MRSA isolate from sputum was susceptible to all the non–β-lactam antimicrobial drugs tested, including erythromycin, clindamycin, ciprofloxacin, tetracycline, kanamycin, and fusidic acid. With established molecular methods, the isolate was found to harbor *SCCmec* type IV (*5*); *lukS* and *lukF*, the genes coding for the 2 subunits of the PVL toxin; and *hlg*, the γ-hemolysin gene (*3*). The genetic background of the isolate was determined by multilocus sequence typing (MLST) (*6*) and sequence typing of the tandem repeat region of protein A gene (*spa* typing) (*7*). Results showed that the isolate belonged to ST30 according to the MLST database (http://saureus.mlst.net), and *spa* typing, analyzed by the Ridom Staphtype software (http://www.ridom.de), indicated a novel *spa* type, to which type 755 was assigned. ST30, 1 of 6 clones more commonly associated with PVL-positive CA-MRSA (*4*), is designated also the southwest Pacific (SWP) clone, because of the area in which it circulates. Recently, the SWP clone has caused CA-MRSA infections in northern European countries (England, Scotland, the Netherlands, Sweden, and Latvia) (*8**,**9*). Molecular analysis suggests that the SWP clone has evolved from a methicillin-susceptible clone of *S. aureus*, termed phage type 80/81, that was pandemic in the 1950s and considered to be unusually virulent and transmissible (*8*). In fact, strains belonging to phage type 80/81 carry the PVL gene and appear to have subsequently acquired methicillin resistance through horizontal transfer of *SCCmec* type IV. The *spa* type of the Italian isolate comprises 7 nucleotide repeats, indicated by XJ4AKAOM in the alphabetical code. This repeat sequence differs from that of the classical SWP clone, indicated by XKAKAOMQ (*8*), by only 1 bp in the second repeat and loss of the last Q repeat. In spite of these differences, the *spa* type is in substantial agreement with the MLST result and indicates that the Italian isolate is either a descendent or a local variant of the SWP clone. The most common clone of CA-MRSA described in Europe is ST80, *spa* type 44. CA-MRSA belonging to ST80 tend to be more antimicrobial drug resistant than isolates belonging to other clones (*4*). Resistance to fusidic acid, typical of ST80, has been proposed as a marker for CA-MRSA in Europe (*10*). In light of our finding, we cannot rely on resistance to fusidic acid to screen for PVL-producing CA-MRSA in our country.

To our knowledge, this is the first report from Italy of necrotizing pneumonia caused by PVL-positive CA-MRSA. The presentation was typically that of a severe pneumonia that occurred in a previously healthy, young adult with no risk factors for MRSA acquisition, as described in other cases (*11*). This is also the first report of a SWP clone isolate in southern Europe; if the strain is circulating in Italy or is occasionally imported from the SWP area, whether our patient acquired it through contact with a foreign contact remains unknown.
