# Correction: Vol. 11, No. 10

**DOI:** 10.3201/eid1111.C21111

**Published:** 2005-11

**Keywords:** errata, erratum, correction

In “Methicillin-resistant *Staphylococcus aureus* Necrotizing Pneumonia,” by Monica Monaco et al., an error occurred on page 1647, in the first full sentence of the third column. The sentence should read “On day 3 of admission, antimicrobial drug therapy was changed to linezolid (600 mg 2 times a day).”

The corrected text appears in the online article at http://wwwnc.cdc.gov/eid/article/11/10/05-0776_article.htm.

We regret any confusion this error may have caused.

